# Effect of crocin on biochemical parameters, oxidative/antioxidative profiles, sperm characteristics and testicular histopathology in streptozotocin-induced diabetic rats

**Published:** 2019

**Authors:** Seyed Mersad Sefidgar, Mahmood Ahmadi-hamedani, Ashkan Jebelli Javan, Reza Narenji Sani, Abbas Javaheri Vayghan

**Affiliations:** 1 *Student Research Committee, Faculty of Veterinary Medicine, Semnan University, Semnan, Iran.*; 2 *Department of Clinical Sciences, Faculty of Veterinary Medicine, Semnan University, Semnan, Iran.*; 3 *Department of Food Hygiene, Faculty of Veterinary Medicine, Semnan University, Semnan, Iran.*; 4 *Department of Pathobiology, Faculty of Veterinary Medicine, Semnan University, Semnan, Iran.*

**Keywords:** Biochemical parameters, Crocin, Oxidative stress index, Sperm characteristics, Streptozotocin, Testicular histopathology

## Abstract

**Objective:**

Chronic hyperglycemia and overproduction of reactive oxygen species (ROS) are strong predictors of the development of reproductive complications of diabetes. The present study was conducted to determine the effects of crocin on biochemical parameters, oxidative stress, and sperm characteristics as well as testes histopathology in diabetic rats.

**Materials and Methods:**

Twenty-four rats were divided into the four groups as follows: control, untreated diabetic and two crocin (40 and 60 mg/kg/day)-treated diabetic groups. Diabetes was induced by injection of a single dose of streptozotocin (STZ, 60 mg/kg). Administration of crocin (intraperitoneally) was started three days after STZ injection and was continued until the 28^th^ day. At the end of the experiment, rats were anesthetized after weighing. Blood samples and epididymal sperm were subsequently collected to measure biochemical parameters (glucose and lipid profile), total oxidant and antioxidant status (TOS and TAS, respectively), oxidative stress index (OSI), and sperm characteristics (count, motility, and viability); also, testes were dissected out for histopathology examination.

**Results:**

Our result indicated that blood glucose, cholesterol, triglyceride, LDL cholesterol levels, as well as TOS, and OSI decreased, but body weight, sperm counts, motility and viability, as well as TAS and HDL levels increased significantly in the crocin-treated diabetic rats (P˂0.05). In testis sections from diabetic rats treated with crocin (40 and 60 mg/kg), seminiferous tubules exhibited normal shape and restoration of testis architecture was observed.

**Conclusion:**

Administration of crocin in the present study, ameliorated blood glucose, lipid abnormalities, oxidative stress, sperm characteristics and testis damage in STZ-diabetic rats.

## Introduction

Diabetes mellitus (DM) is a prevalent metabolic disorder which is characterized by chronic hyperglycemia resulting from defective insulin production, insulin response, or both (Jain and Jangir, 2014 ▶). Based on the fact that impotency, retrograde ejaculation, and hypogonadism are the most important indicators of male infertility, numerous studies indicated that DM in both diabetic men and animals (Shi et al., 2017 ▶). The Undesirable effects of DM on male sexual functions may occur indirectly through the hormonal changes of the hypothalamic-pituitary-gonadal axis, or directly due to the effect of insulin on the testes and sperm cells (Schoeller et al., 2012 ▶). Insulin production by the testes and sperm cells and its expression in the testes are affected by DM (Gomez et al., 2009 ▶). Glucose metabolism plays an important role in the process of spermatogenesis and its disorder in DM, both in men and in knockout mice dramatically reduces spermatogenesis, and increases germ cell depletion and Sertoli cell vacuolization (Bruning et al., 2000 ▶; Bacceti et al., 2002 ▶). Several studies reported harmful effects of DM on male reproductive system including, reduced semen volume, decreased motility and viability of sperm, chromatin quality and forming the nuclear DNA fragment (Agbaje et al., 2007 ▶; Kilarkaje et al., 2015 ▶; Cavallini, 2006 ▶). Oxidative stress was fully established as a contributing factor to the pathogenesis of reproductive defects in DM. Increased production of free radicals in most tissues in chronic hyperglycemia results from glucose self-oxidation, protein glycosylation, activation of intracellular hexosamine pathways and advanced glycolysis (Sifuentes-Franco et al., 2017 ▶). Chronic hyperglycemia and the generation of reactive oxygen species (ROS) are strong predictors of the development of diabetic complications (Niedowicz and Daleke, 2005 ▶). Macrophages are activated by hyperglycemia and consequently increase the proinflammatory cytokines in the testes. Free radicals and ROS increase with chronic inflammation, but decreased levels of enzymatic antioxidants were determined in diabetic patients (Evans et al., 2002 ▶). There are two kinds of free radical scavenging systems to reduce destructive effects of these radicals in cells: enzymatic (catalase (CAT), superoxide dismutases (SOD), and glutathione peroxidase (GPX)) and nonenzymatic (glutathione (GSH), α-tocopherol, vitamin C, and urate) free radical scavenging systems (Samarghandian et al., 2016 ▶). 

Laboratory animals are ideal models for studying diabetes, because they have testicular abnormalities similar to those observed in diabetic patients. Streptozotocin (STZ) administration (60 mg/kg i.p.) to rats can lead to significant reductions in the weight of the testes and epididymis, testosterone levels and diameters of the seminiferous tubules and damage Sertoli cell (Xu et al., 2014 ▶).

Recent growing evidence suggests that plant products with potent antioxidant properties can be used as an alternative medicine to improve the management of diabetes complications (Salahshoor et al., 2016 ▶). 

Saffron (*Crocus sativus* L.) as a perennial herb without stem of the Iridaceae family, is cultivated in Iran, Europe, Turkey, Central Asia, India, China and Algeria. Three major chemical constituents of saffron including safranal (volatile oil), picrocrocin (bitter principle) and crocin (colored component) have a potent antioxidant capacity (Rios et al., 1996 ▶). Crocin as one of the substances of saffron, has a very diverse range of pharmacological effects such as antitumor (Samarghandian and Borji, 2014 ▶), antinociceptive, anti-inflammatory (Hosseinzadeh and Younesi, 2002 ▶), antioxidant (Farahmand et al., 2013 ▶), antidepressant (Samarghandian et al., 2013), hypolipidemic (Sheng et al., 2006 ▶), memory inhancing (Heidari et al., 2017 ▶), fertility improving and sexual copulation strengthening (Hosseinzadeh et al., 2005 ▶), spermatogenesis ameliorative effects (Bayatpoor et al., 2018 ▶; Sapanidou et al., 2015 ▶), and it could attenuate the toxic effects of cyclophosphamide on testis (Potnuri et al., 2018 ▶; Bakhtiary et al., 2014 ▶), improve *in vitro* maturation of mouse oocytes (Mokhber Maleki et al., 2016 ▶), and neutralize anemia complications on testicular parameters (Kalantari hesari et al., 2015 ▶). 

The antioxidant potency of crocin as well as its ability to neutralize free radicals were investigated (Bolhassani et al., 2014 ▶). Insulin-dependent diabetes affects sperm parameters in men, but so far no specific study has investigated the effects of crocin on reproductive, biochemical and oxidative parameters in diabetic rats. Therefore, this study was conducted to determine the effects of crocin on plasma levels of glucose, lipids, oxidative stress markers and evaluate the changes in sperm parameters (count, motility and viability) and testes histopathology in diabetic rats.

## Materials and Methods

**Chemicals**

STZ, crocin, acetic acid, and sodium citrate were purchased from Sigma-Aldrich chemical (St. Louis, USA). Cholesterol, Triglyceride, LDL and HDL-cholesterol diagnostic kits were obtained from Ziest Chem Diagnostics, Inc. (Tehran, Iran). All the other chemical used in the present study were of analytical grade.

**Animals**

Twenty-four healthy adult male Wistar rats (weighing 200-250 g) were obtained from the experimental animal care center of Semnan University. One week before the start of the experiments, rats were housed in their own cages under standard conditions (21±3°C; 12 hr/12 hr light/dark cycle) with free access to food and water. These conditions were maintained constant until the end of the experiments. This study was conducted at Semnan University of Veterinary Sciences, Semnan, Iran, and protocols were approved by the Ethical Research Committee of Semnan University of Veterinary Sciences. 

**Induction of diabetes by STZ**

In order to induce diabetes in rats, the diabetic groups were received a single intraperitoneal (i.p.) injection of STZ (diluted in freshly prepared 0.01 M citrate buffer, pH 4.5) at a dose of 60 mg/kg on the first day of the experiment (i.e. day 1). The fasting blood glucose was measured 72 hr after STZ injection by using Accu-Check Glucometer, (Roche). Rats with blood glucose values higher than 250 mg/dl were considered diabetic.

**Experimental design**

Twenty-four male Wistar rats were randomly divided into four experimental groups (six animals in each) as follows: control (C) group; diabetic (D) group; diabetic group treated with crocin (40 mg/kg/day) (D+C40) and diabetic group treated with crocin (60 mg/kg/day) (D+C60) (Mohammadi et al., 2018; Ahmadi et al., 2017). In the treatment groups (D+C40 and D+C60), crocin (i.p.) was administrated daily from day 3 for 4 weeks (Samarghandian et al., 2016). The control and untreated diabetic groups received normal saline (i.p.) as vehicle. Blood glucose level and body weights were measured at two time points, before the start of the experiment (as the initial blood glucose and the initial body weight), and the last day of the experiment (28^th^ day; as the final blood glucose and final body weight). At the end of the experiment on day 28, animals were killed under anesthesia using chloroform and blood was subsequently collected from the heart of the rats and epididymis and testes were removed. Blood was allowed to clot at room temperature and then, centrifuged at 3000 rpm for 10 min to assess glucose, and lipid profiles, and total oxidant/antioxidant status and oxidative stress index. Epididymis and testes were removed for sperm analysis and histopathological examination, respectively.

**Measurement of blood glucose**

Initial glucose levels were measured by an Accu-Check One Touch glucometer (Accu-Check Glucometer, Roche) using rat tail vein blood. Final glucose concentrations in 10 µL serum were determined by commercial diagnostic kits (Pars Azmoon, Tehran, Iran) using an automatic analyzer (Mindray, BS-120, Shanzhen 518057, P.R. China).

**Evaluation of serum lipid profile**

Total cholesterol (TC), triglyceride (TG), low-density lipoprotein cholesterol (LDL-C), and high-density lipoprotein cholesterol (HDL-C) concentrations in 30 µL serum were measured by enzymatic colorimetric methods with commercial kits (Pars Azmoon, Tehran, Iran) using an automatic analyzer (Mindray, BS-120, Shanzhen 518057, P.R. China).

**Evaluation of total oxidant status**

Total oxidative status (TOS) of 350 µL serum samples was evaluated using the method described by Erel (2005) ▶. According to this method, the ferrous ion-o-dianisidine complex is oxidized to ferric ion by oxidants present in serum samples. This reaction is accelerated by abundant glycerol molecules in the medium. The ferric ions in an acidic medium make a colored complex with xylenol orange. The color intensity, which is proportional to the total amount of oxidant molecules in the sample, can be measured spectrophotometrically. Calibration of this assay was carried using hydrogen peroxide and the results are expressed as micromoles of hydrogen peroxide equivalents per liter (μmol H_2_O_2_ equivalents/L).

**Evaluation of total antioxidant status**

Total antioxidant status (TAS) of 50 µL serum samples was evaluated using the method reported by Erel (2004) ▶. This method is generally based on the ability of antioxidants in the samples to reduce or inhibit oxidized products produce during the assay. After incubation of ABTS (2,2’-azino-bis (3-ethylbenz-thiazoline-6-sulfonic) with hydrogen peroxide a green-blue ABTS radical (ABTS^++^) is made which has maximum absorptions at 650, 734 and 820 nm. Reduction of ABTS^++^ by antioxidants affects this color production, which is proportional to their concentrations. The assay is commonly calibrated using Trolox, a water-soluble analogue of vitamin E, and the results are expressed as mmol Trolox equivalents per liter (mmol Trolox/l).

**Oxidative stress index**

The oxidative stress index (OSI) value is expressed as the percentage of the TOS to TAS (μmol H_2_O_2_ equivalents/ mmol Trolox equivalents) (Dokuyucu et al. 2014 ▶).

**Epididymal sperm analysis**

The caudal area of the right epididymis from each rat was excised and preserved in a warmed (37°C) petri dish containing 2 ml normal saline. The sperms were allowed to swim-out from the epididymis into the medium. After 15 min, the epididymis was removed and the suspension was gently shaken to become homogenized. The quality of sperm suspension was analyzed according to the World Health Organization (WHO) guidelines (World Health Organization, 1996) and evaluated by a Neubauer hemocytometer chamber in terms of total number, motility and viability of sperms. The number of spermatozoa was calculated as follows: 200 μL of the sperm suspension was diluted (1:5) with normal saline which contained a few drops of formalin 40% to kill sperm. Then 10 μL of the suspension was transferred into a hemocytometer using a sterile Pasteur pipette (Germany). Number of spermatozoa/ml of sperm suspension of caudal epididymis=total number of spermatozoon in four small corner squares×125×10^2 ^(Saber et al., 2016 ▶). Sperm motility was assessed visually by a light microscope (Olympus, Japan) at 400x magnification. Briefly, a drop of sperm suspension and covered by a pre-warmed glass cover slide at 37°C, and the percentage of motile sperm was determined by counting more than 200 spermatozoa in several random fields (Saber et al., 2016 ▶). Sperm viability was evaluated according to the method previously described by Saber et al., (2016) ▶. In brief, one drop of sperm suspension was mixed with two drops of 1% aqueous bluish eosin (Merck, Darmstadt, Germany) solution. After 30 sec, three drops of 10% aqueous nigrosin (Merck, Darmstadt, Germany) were added to the mixture and the mixture was examined under a light microscope (at 1000x magnification). Dead spermatozoa stained pink or red, while live spermatozoa remained colourless. The percentage of live spermatozoa was calculated by counting at least 200 sperms in 10 microscopic fields (Sakhaee et al., 2016 ▶). 

**Histopathological examination of the testis**

The left testis was fixed in 10% neutral buffered formalin, dehydrated using ethanol and embedded in paraffin wax. The microscopic sections (5 μm) were prepared and stained with hematoxylin and eosin (H&E). The specimens were mounted using Entellan and observed under a light microscope (Olympus, Lake Success, NY, USA) and the imaged. One of the authors, Dr. A. Javaheri Vayghan who observed the histology slides, was blinded to the treatment groups.

**Statistical analysis**

All data were analyzed using SPSS statistical software version 19. Values are presented as mean±standard deviation (SD) and Shapiro-Wilk test was used to determine the normal distribution of the data. The mean of the studied variables was compared between the untreated diabetic and crocin-treated diabetic groups with the control group using one-way analysis of variance (ANOVA) and Tukey's post *hoc test*. A p<0.05 was considered statistically significant.

## Results

**Body weight**

There was a significant (p<0.001) body weight loss in the untreated diabetic rats in comparison with the control rats, after 4 weeks (experimental period). However, body weight in rats treated with crocin (40 and 60 mg/kg) significantly (p<0.001) increased compared to the untreated diabetic rats, at the end of the experimental period. No significant difference was observed between the diabetic group treated by highest crocin dose (60 mg/kg/day) and the control group after 4 weeks experimental period (Table 1).

**Table 1:** Body weight in control (C), untreated diabetic rats (D), treated diabetic rats–crocin (40 mg/kg) (D+C40) and treated diabetic rats–crocin (60 mg/kg) (D+C60) during 4 weeks of study

Days	0	7	14	21	28
C					
Body weight (g)	207.16 ± 11.30	220.83 ± 12.00	229.66 ± 13.72	245.66 ± 14.05	262.16 ± 24.12
D					
Body weight (g)	205.00 ± 13.78	196.83 ± 12.49^a^	189.00 ± 15.20^a^	182.00 ± 10.07^a^	173.00 ± 11.71^a^
D + C40					
Body weight (g)	196.60 ± 4.77	202.40 ± 7.02	211.20 ± 13.29	225.40 ± 15.14^b^	231.80 ± 14.66^b^
D + C60					
Body weight (g)	207.16 ± 11.21	217.50 ± 12.46^b^	228.66 ± 14.23^b^	239.33 ± 14.90^b^	246.33 ± 16.54^b^

Data were represented as mean±SD for six rats in each group.

a Significantly different from control rats (; p<0.05).

b Significantly different from untreated diabetic rats (; p<0.05).

**Blood glucose levels**

**Figure 1:**
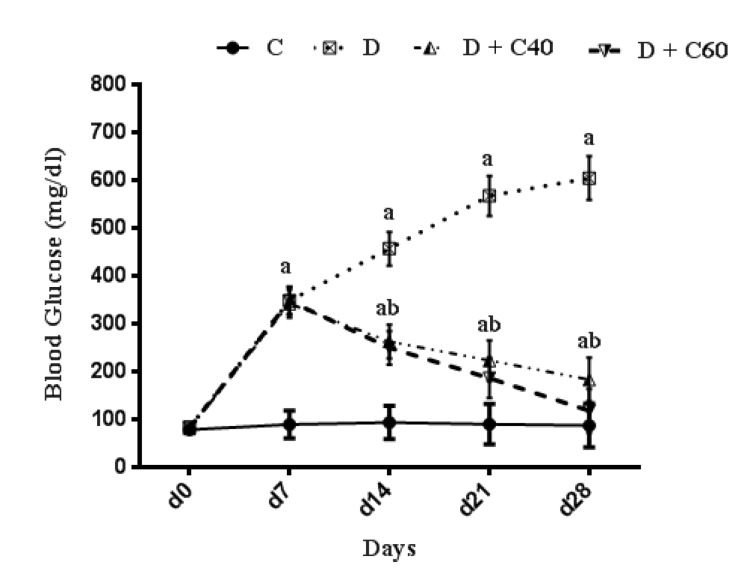
Effect of crocin on Blood Glucose Level (mg/dl). Control (C), untreated diabetic rats (D), treated diabetic rats–crocin (40 mg/kg) (D+C40) and treated diabetic rats–crocin (60 mg/kg) (D+C60) during 4 weeks of study (n=6, for each group). Values are expressed as mean±SD. Significance differences of groups when compared to control group: a; p<0.05. Significant differences of groups when compared to untreated diabetic group: b; p<0.05

Significant (p<0.001) hyperglycemia was determined in diabetic rats compared to the control rats (Figure 1). Blood glucose in STZ diabetic rats treated by crocin (40 and 60 mg/kg/day) significantly decreased at week 2 (p<0.05 and p<0.05, respectively), 3 (p<0.05 and p<0.05, respectively) and 4 of the study as compared to untreated diabetic rats (p<0.05 and p<0.01, respectively) (Figure 1). In diabetic rats receiving the highest dose of crocin (60 mg/kg/day), hyperglycemia began to significantly decline from the 3rd week of treatment (p<0.001) (Figure 1). As shown in Figure 1, blood glucose levels in the diabetic group treated with crocin 60 mg/kg was almost close to the control group, 4 weeks after initiation of the study. 

**Modification of serum lipid profile by crocin**

**Figure 2:**
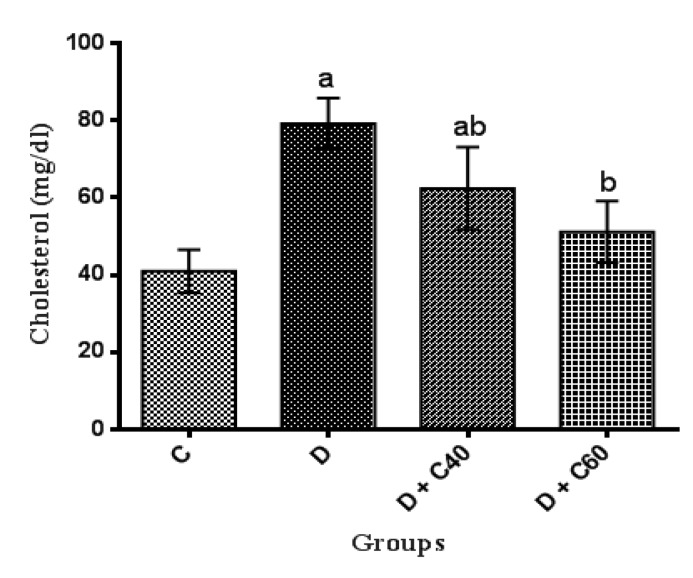
Effect of crocin on serum cholesterol level. Control (C), untreated diabetic rats (D), treated diabetic rats–crocin (40 mg/kg) (D+C40) and treated diabetic rats–crocin (60 mg/kg) (D+C60) during 4 weeks of study (n=6, for each group). Values are expressed as mean±SD. Significance differences of groups when compared to control group: a; p<0.05. Significant differences of groups when compared to untreated diabetic group: b; p<0.05

**Figure 3:**
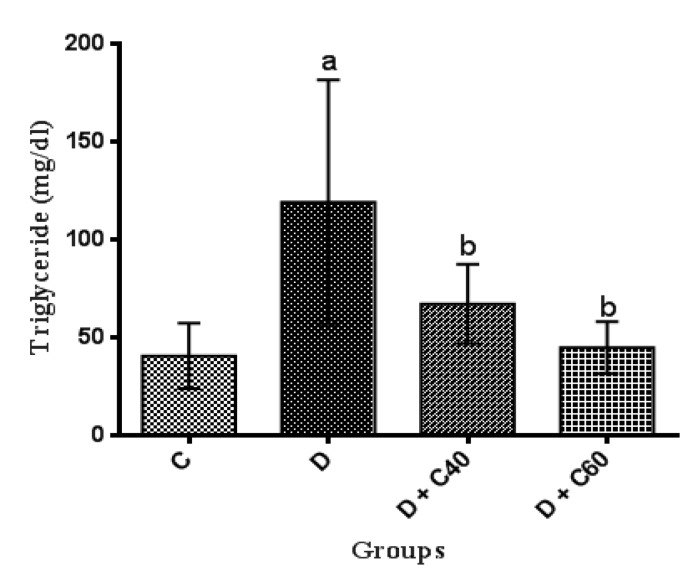
Effect of crocin on serum triglyceride level. Control (C), untreated diabetic rats (D), treated diabetic rats–crocin (40 mg/kg) (D+C40) and treated diabetic rats–crocin (60 mg/kg) (D+C60) during 4 weeks of study (n=6, for each group). Values are expressed as mean±SD. Significance differences of groups when compared to control group: a; p<0.05. Significant differences of groups when compared to untreated diabetic group: b; p<0.05

**Figure 4:**
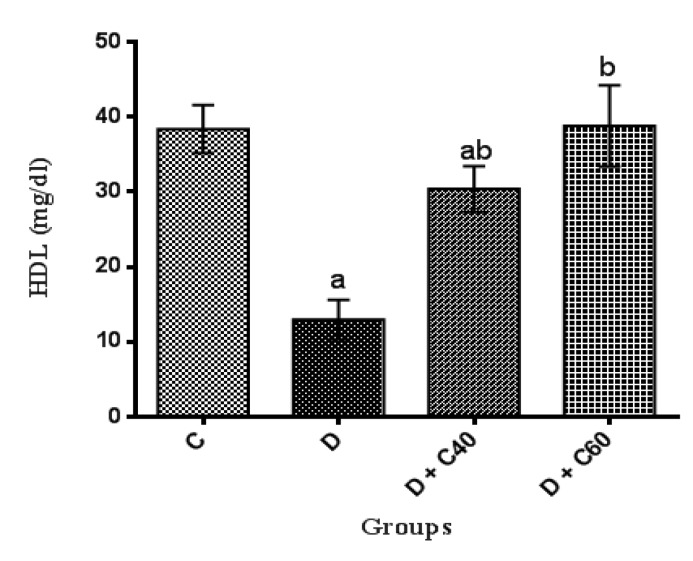
Effect of crocin on serum HDL-cholesterol level. Control (C), untreated diabetic rats (D), treated diabetic rats–crocin (40 mg/kg/day) (D+C40) and treated diabetic rats–crocin (60 mg/kg) (D+C60) during 4 weeks of study (n=6, for each group). Values are expressed as mean±SD. Significance differences of groups when compared to control group: a; p<0.05. Significant differences of groups when compared to untreated diabetic group: b; p<0.05

**Figure 5:**
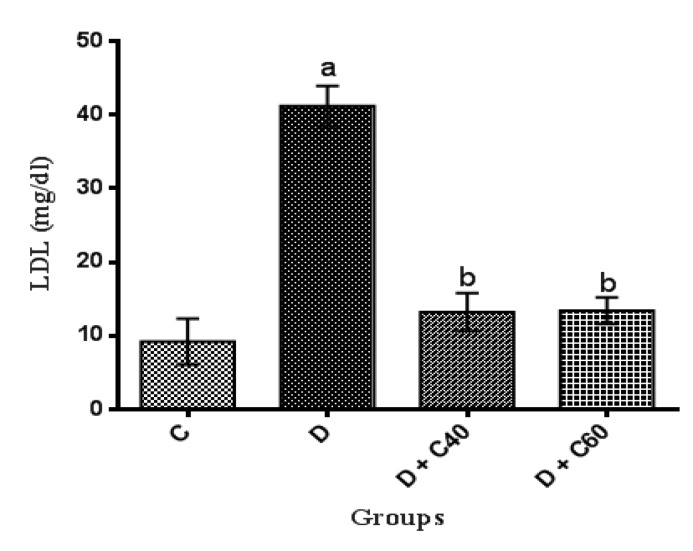
Effect of crocin on serum LDL-cholesterol level. Control (C), untreated diabetic rats (D), treated diabetic rats–crocin (40 mg/kg) (D+C40) and treated diabetic rats–crocin (60 mg/kg) (D+C60) during 4 weeks of study (n=6, for each group). Values are expressed as mean±SD. Significance differences of groups when compared to control group: a; p<0.05. Significant differences of groups when compared to untreated diabetic group: b; p<0.05

Serum levels of TC, TG and LDL-C significantly (p<0.01) increased and HDL-C significantly (p<0.01) decreased, in untreated diabetic group compared to the control group (Figures 2, 3, 4 and 5). After the 4-week experimental period, crocin decreased the serum levels of TC, TG and LDL-C but increased serum levels HDL-C (p<0.05) (Figures 2, 3, 4 and 5). The diabetic group treated with the highest crocin dose (60 mg/kg/day) did not show a significant difference in lipid profile compared to the control group (Figures 2, 3, 4 and 5).

**Modification of serum total oxidant/antioxidant status and oxidative stress index**

**Figure 6:**
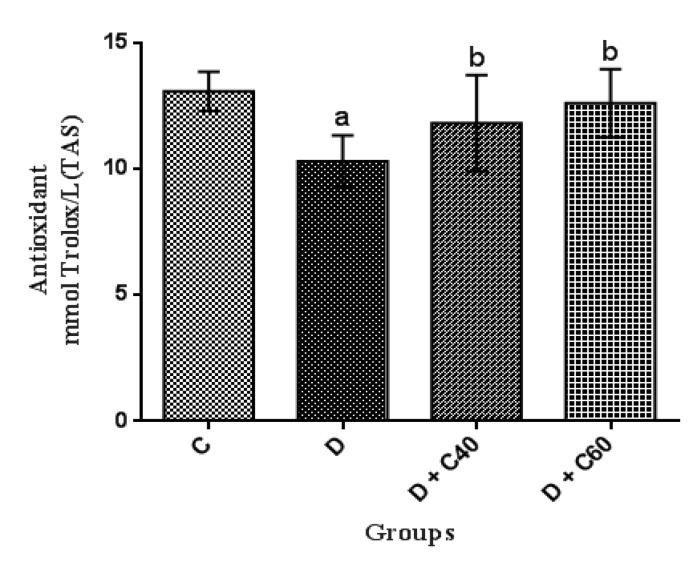
Effect of crocin on total antioxidant status (TAS). Control (C), untreated diabetic rats (D), treated diabetic rats–crocin (40 mg/kg) (D+C40) and treated diabetic rats–crocin (60 mg/kg) (D+C60) during 4 weeks of study (n=6, for each group). Values are expressed as mean±SD. Significance differences of groups when compared to control group: a; p<0.05. Significant differences of groups when compared to untreated diabetic group: b; p<0.05

**Figure 7:**
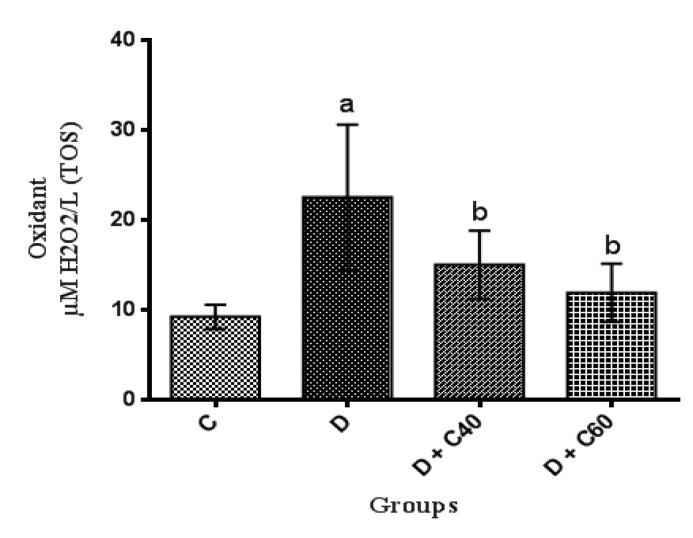
Effect of crocin on total oxidant status (TOS). Control (C), untreated diabetic rats (D), treated diabetic rats–crocin (40 mg/kg) (D+C40) and treated diabetic rats–crocin (60 mg/kg) (D+C60) during 4 weeks of study (n=6, for each group). Values are expressed as mean±SD. Significance differences of groups when compared to control group: a; p<0.05. Significant differences of groups when compared to untreated diabetic group: b; p<0.05

**Figure 8:**
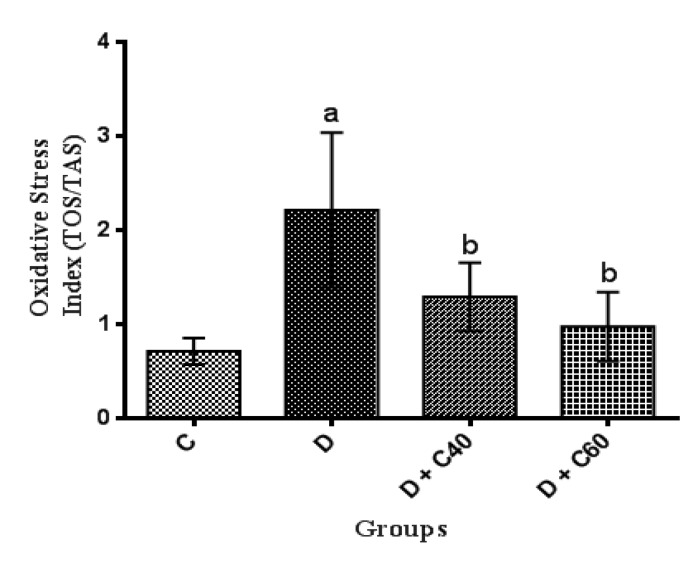
Effect of crocin on oxidative stress index (TOX/TAS). Control (C), untreated diabetic rats (D), treated diabetic rats–crocin (40 mg/kg) (D+C40) and treated diabetic rats–crocin (60 mg/kg) (D+C60) during 4 weeks of study (n=6, for each group). Values are expressed as mean±SD. Significance differences of groups when compared to control group: a; p<0.05. Significant differences of groups when compared to untreated diabetic group: b; p<0.05

Diabetic rats showed significant increases (p<0.01) in serum total oxidant status and oxidative stress but significant decreases (p<0.01) in serum total antioxidant status compared to the control rats (Figure 6). Diabetic rats treated with crocin (40 and 60 mg/kg/day) had a significant (p<0.05) decrease in serum total oxidant status (Figure 7) and oxidative stress (Figure 8) but a significant increase (p<0.05) in serum total antioxidant status: crocin 60 mg/kg produced a more marked decrease in the level of these parameters compared to crocin 40 mg/kg (Figure 6). 

**Effect of crocin on sperm counts, motility and viability**

**Figure 9:**
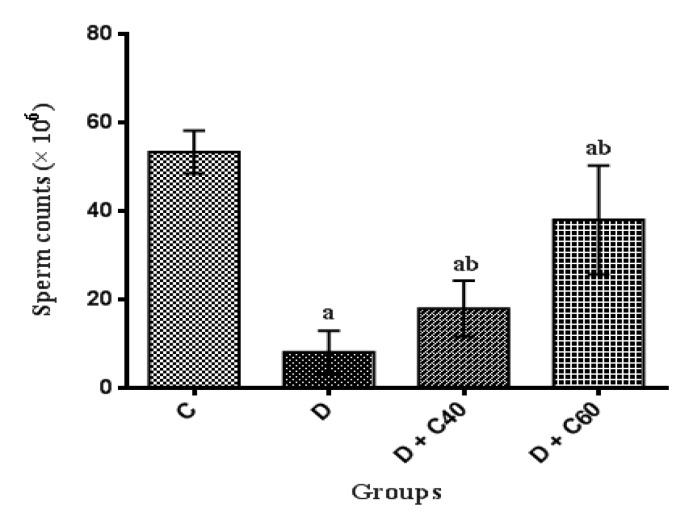
Effect of crocin on Sperm Counts (×10^6^). Control (C), untreated diabetic rats (D), treated diabetic rats–crocin (40 mg/kg) (D+C40) and treated diabetic rats–crocin (60 mg/kg) (D+C60) during 4 weeks of study (n=6, for each group). Values are expressed as mean±SD. Significance differences of groups when compared to control group: a; p<0.05. Significant differences of groups when compared to untreated diabetic group: b; p<0.05

**Figure 10:**
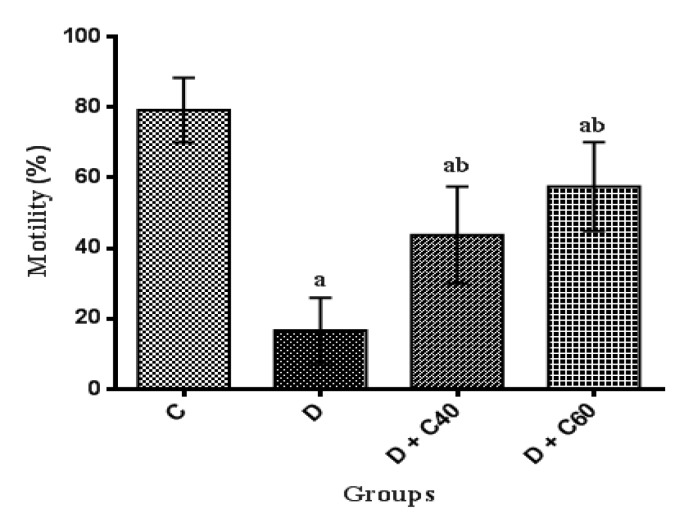
Effect of crocin on Motility (%). Control (C), untreated diabetic rats (D), treated diabetic rats–crocin (40 mg/kg/day) (D+C40) and treated diabetic rats–crocin (60 mg/kg/day) (D+C60) during 4 weeks of study (n=6, for each group). Values are expressed as mean±SD. Significance differences of groups when compared to control group: a; p<0.05. Significant differences of groups when compared to untreated diabetic group: b; p<0.05

**Figure 11:**
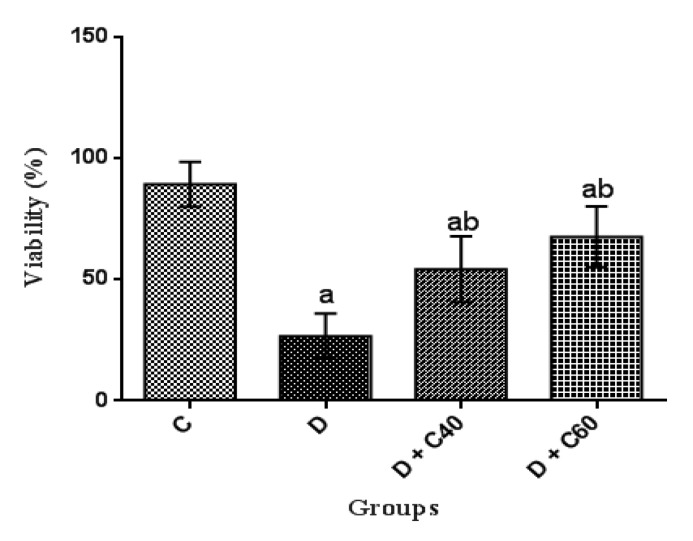
Effect of crocin on Viability (%). Control (C), untreated diabetic rats (D), treated diabetic rats–crocin (40 mg/kg) (D+C40) and treated diabetic rats–crocin (60 mg/kg) (D+C60) during 4 weeks of study (n=6, for each group). Values are expressed as mean±SD. Significance differences of groups when compared to control group: a; p<0.05. Significant differences of groups when compared to untreated diabetic group: b; p<0.05

Sperm counts, motility and viability were significantly (p<0.01) decreased in the diabetic rats (8.06±4.85, 16.66±9.31 and 26.66±9.31, respectively) compared to the control rats (53.33±4.84, 79.16±9.17 and 89.16±9.17, respectively) (Figures 9, 10 and 11). Crocin administration (40 and 60 mg/kg/day) significantly (p<0.05) increased sperm counts (18.24±7.05 and 38±1.23, respectively), motility (45±15 and 57.5±12.55, respectively) and viability (55±15 and 67.5±12.55, respectively) in diabetic-treated groups compared to diabetic-untreated group during the experimental period (Figures 9, 10 and 11). Furthermore, sperm counts, motility and viability in diabetic rats treated with crocin (60 mg/kg/day) were significantly higher than those in rats treated with crocin (40 mg/kg/day) (Figures 9, 10 and 11).

**Effect of crocin on seminiferous tubules**

**Figure 12:**
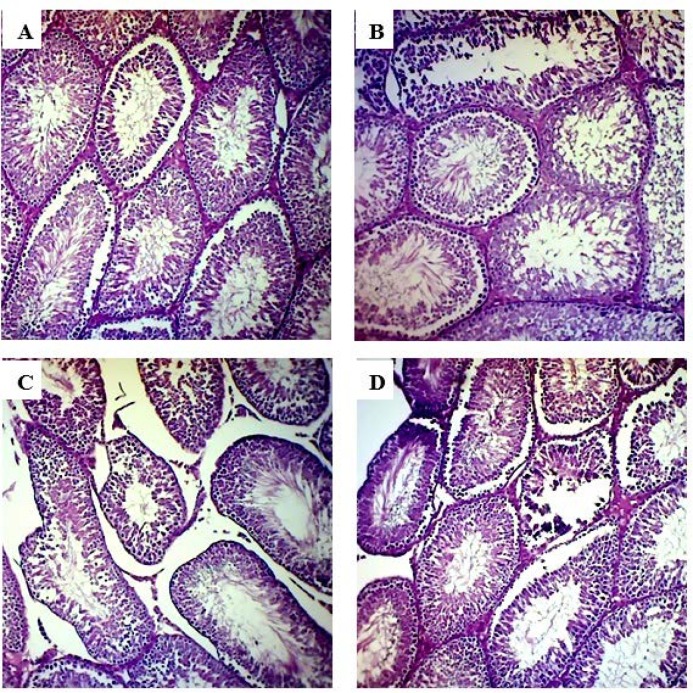
Histopathological study of testicular tissue and effect of crocin in testicular tissue in diabetic rats. H&E staining with magnification at X100.

The histopathologic architecture of testis in diabetic rats compared to the control rats exhibited severe irregularities in the shape of seminiferous tubules, including an abnormal attachment of germ cells and cellular necrosis, a decrease in the thickness epithelium of the germinal layer, and a marked increase in the connective tissue of interstitial space (Figures 12A and 12B). In crocin-40 and 60 mg/kg treated diabetic rats, a relative improvement of these parameters was observed in comparison to the untreated diabetic group, but these improvements did not reach the control group values (Figures 12C, and 12D). 

A: Healthy control rats with regular seminoferous tubules, intact germinal epithelium and normal attachment of germ cells. B: Diabetic control rats with irregular seminoferous tubules, disrupted germinal epithelium and marked depletion of germ cells. C: Diabetic rats treated with crocin 40 mg/kg showed mild disruption of germinal epithelium and relatively moderate deplation of germ cells in seminoferous tubules. D: Diabetic rats treated with crocin 60 mg/kg with relative restoration of the testicular architecture and normal seminoferous tubules.

## Discussion

Diabetes mellitus (DM), as a major health problem, affects the quality of life of diabetic individuals. Epidemiological, experimental studies and clinical evidences showed that DM affects the reproductive system. Diabetes-induced male reproductive system damage is one of the major secondary complications of DM in animals and humans (Shi et al., 2017 ▶). DM has devastating effects on reproduction, including causing histological damage to the epididymis and testis (Ghanbari et al., 2015 ▶), and decreasing semen volume (Adedara et al., 2015 ▶), sperm counts, motility, and morphological change (Afifi et al., 2015 ▶; Saumya & Basha, 2016 ▶) and reducing the size of the seminiferous tubules (Rashid and Sil, 2015 ▶). Despite the fact that several medicinal plants were introduced to improve DM-induced reproduction dysfunctions in various traditional and folk medicine worldwide, no specific treatment for reproductive issues of diabetic patients has been developed. Thus, the use of medicinal plants to overcome the reproductive implications of DM is considered an ideal approach (Shi et al., 2017 ▶). 

STZ-induced DM in rats is commonly used as an appropriate model for the study of testicular dysfunction. Crocin has long been considered for many pharmacological properties including anti-inflammatory (Hosseinzadeh and Younesi, 2002 ▶), antioxidant (Bolhassani et al., 2014 ▶), antitumor (Samarghandian and Borji, 2014 ▶) and anti-infertility (Hosseinzadeh et al., 2005 ▶) activities, in the Iran traditional medicine. In this research, we showed the effectiveness of crocin in improving testicular spermatogenesis dysfunction in diabetic male rats. 

The present study demonstrated us the daily administration of crocin (40 and 60 mg/kg) reduced blood glucose levels compared to the untreated diabetic group. The onset of the decrease in blood glucose levels in crocin-treated rats occurred from day 14 of the study, which this reduction was significantly increased every day until the end of the study (day 28). The reduction of blood glucose observed in this study, was consist with previous studies (Asri-Rezaei et al., 2015 ▶; Samarghandian et al., 2016 ▶). The reduction in blood glucose after 28 days in diabetic rats treated with crocin 60 mg/kg was consistent with data reported by Bayatpour et al. (2018) ▶, which demonstrated that administration of crocin 20 mg/kg to diabetic rats for 60 days, reduces blood glucose. Although the precise mechanism of blood glucose lowering effect of crocin is beyond the scope of this study, it was shown that crocin increases insulin sensitivity in glucose metabolism through activation of both insulin-dependent (phosphatidylinositol-3 kinase/protein kinase B and mammalian target of rapamycin; PI 3-kinase/AKT and mTOR) and insulin-independent (5-AMP-activated protein kinase/acetyl-COA carboxylase (AMPK/ACC) and mitogen-activated protein kinases (MAPKs)) pathways (Kang et al., 2012 ▶). In addition, crocin injection improved STZ-induced abnormalities in serum lipid profiles, including TC, TG, LDL-C, and HDL-C, which may be due to increased hepatic and adipose tissue uptake of serum lipid or decreased hepatic synthesis of cholesterol and fatty acids (Shirali et al., 2013 ▶). STZ-injected rats exhibited significant weight loss compared to the control rats, while treatment with crocin significantly increased body weight in diabetic groups at the end of the study period. Together, treatment of diabetic rats with crocin reduced blood glucose levels and improved body weight and lipid profile. Furthermore, treatment of diabetic rats with crocin significantly improved serum total antioxidant capacity, attenuated serum total oxidant status as well as stress oxidative index compared to the untreated diabetic rats. These data are consistent with previously reported findings on the use of crocin as an antioxidant for treatment of various diseases (Bors et al., 1984 ▶; Tubaro et al., 1996 ▶; Kampa et al., 2002 ▶; Hosseinzadeh et al., 2009 ▶; Bathaie et al., 2011 ▶; Bountagkidou et al., 2012 ▶). A major contributor to the complications and pathogenesis of diabetes and its progression is overproduction of oxygen free radicals (oxidative stress) caused by hyperglycemia. Evidence from several studies showed that STZ induces the formation of oxygen free radicals, resulting in complications of diabetes and its progression (Yazdanparast et al., 2007 ▶). Extensive studies have suggested that the biological activities of crocin is probably due to the presence of sugars attached to the crocetin moiety (Khazdair et al., 2015 ▶). A study conducted by Ordoudi et al. (2009) ▶ showed that the bioactive molecule of crocin counteracts free radicals and enhances the body’s natural enzymatic and non-enzymatic antioxidant defense system. The results of another study also suggested that treatment of diabetic rats with crocin regulates the expression of antioxidant-related genes and consequently, the oxidative status (Samarghandian et al., 2016 ▶).

In untreated rats with STZ-induced diabetes, a significant decrease in sperm count, motility and viability was observed compared to control rats. Lack of insulin in STZ-induced diabetic rats affects serum levels of Follicular Stimulating Hormone (FSH), Luteolysis Hormone (LH) and testosterone concentrations due to the reduction of Sertoli and Leydig cells. The main mechanism undelying testopathy-induced by STZ are NADPH oxidase activation in germ cells (a major source of ROS generation in the testis) and increasing the transcription of endothelin1 (ET-1, a main factor for activating NADPH oxidase), ET converting enzyme (ECE) and ET receptors (Kwak et al., 2003 ▶; Kanter et al., 2012 ▶). Evidence suggested that hyperglycemia elevates mitochondrial glucose oxidation and subsequently, a large amount of superoxide and other free radicals are released into the cytoplasm. Therefore, the death of germ cells and the spermatogenesis defect occur due to the excessive generation and accumulation of oxygen free radicals in the mitochondria (Rajender et al., 2010 ▶). STZ-diabetic rats treated with crocin 40 and 60 mg/kg showed significant improvements in sperm count, motility and viability compared to untreated diabetic rats. These results coincided with the findings of Asadi et al., (2014) ▶; Salahshoor et al., 2016 ▶ and Bahojb Soldozi et al., (2018) ▶. The part of the ameliorative effects of crocin on the spermatogenesis process is related may be mediated by a reduction in blood glucose in diabetic rats (Bayatpoor et al., 2018 ▶). Improving the sperm quality after crocin administration may be associated with an increase in the expression of antioxidant genes (Hosseinzadeh et al., 2009 ▶). It seems that the beneficial effects of crocin on sperm parameters in diabetic rats are partly due to the oxygen free radicals (such as hydroxyl radical) scavenging properties, antioxidant and anti-apoptotic activities (Hosseinzadeh et al., 2009 ▶), as well as crocin effects on the biosynthesis of male sex hormones like testosterone in the testis (Bakhtiary et al., 2014 ▶). Many scholars indicated that crocin protects spermatozoa from ROS by activating non-enzymatic antioxidant system (Ding et al., 2016 ▶). 

Similar to previous studies, our investigation demonstrated that diabetes induces a decrease in the diameter of the seminiferous tubules (Salahshoor et al., 2016 ▶). However, administration of crocin in diabetic groups, especially at the dose of 60 mg/kg, significantly increased seminiferous tubules diameter compared to the untreated diabetic groups. In this regard, the study conducted by Bayatpoor et al. (2018) ▶ revealed that the use of crocin during diabetes prevents structural and functional damage to the male reproductive system and greatly improved the spermatogenesis process. It is worth mentioning that the basement membrane thickness of seminiferous tubules increases due to diabetes, which in turn reduces spermatogenesis (Guneli et al., 2008 ▶). The synthesis of basement membrane compounds such as collagen, fibronectin and laminin enhances and basement membrane thickness increases with increasing blood glucose (Rajasekaran et al., 2005 ▶), and anti-oxidant compounds can decrease the volume of the epithelium of the tubules (Salahshoor et al., 2016 ▶). Samarghandian et al. (2014) ▶ demonstrated that safranal as one of the phenolic components of saffron as well as crocin inhibited oxidative damage induced by hyperglycemic in the brain of old male rats. They suggested that safranal is able to cross the blood-brain barrier (BBB). Given that the BBB and blood-testis barrier (BTB) is considered the tightest blood-tissue barriers of the mammalian body, these components are not only able to cross BBB, but also they cross BTB.

In conclusion, crocin 60 mg/kg ameliorated spermatogenic defects in male rats with STZ-induced diabetes. Crocin significantly enhanced total antioxidant status, suppressed total oxidant status, improved sperm characteristics and histopathological architecture. Therefore, the results of this study validated the use of crocin as a potent sperm protective compound for treatment of diabetes mellitus and its reproductive complications. However, more extensive research is needed to elucidate the exact mechanism through which crocin improves the reproductive complications of diabetes. The results of this study clearly showed that crocin 60 mg/kg improves most of the reproductive complications of DM.
